# The Accuracy of Jaws Repositioning in Bimaxillary Orthognathic Surgery with Traditional Surgical Planning Compared to Digital Surgical Planning in Skeletal Class III Patients: A Retrospective Observational Study

**DOI:** 10.3390/jcm9061840

**Published:** 2020-06-12

**Authors:** Martina Barone, Alberto De Stefani, Ugo Baciliero, Giovanni Bruno, Antonio Gracco

**Affiliations:** 1 Department of Neuroscience, School of Dentistry, University of Padua, 35100 Padua, Italy; martinabarone4@gmail.com (M.B.); giobruno93@gmail.com (G.B.); antoniogracco@gmail.com (A.G.); 2 Maxillofacial Surgery Complex Unit of San Bortolo Hospital of Vicenza (Italy), 36100 Vicenza, Italy; baciliero@studiobaciliero.com

**Keywords:** bimaxillary orthognathic surgery, surgical planning, traditional surgical planning, digital surgical planning, surgical simulation, skeletal class III

## Abstract

Background: Technological progress has led to the transition to digital methods to perform surgical planning and to obtain surgical splints with CAD/CAM technologies. The present study aimed to compare the accuracy of jaw repositioning in bimaxillary orthognathic surgery using traditional and digital surgical planning in skeletal class III patients. Methods: This study included 60 skeletal class III patients divided into two groups based on the method used to perform surgical planning: traditional (T, *n* = 30) and digital (D, *n* = 30). For each patient, a 2D presurgical Visual Treatment Objective (VTO) was prepared and the outcome of the surgery was compared with that planned by using determined cephalometric measurements (ANB, SNA, SNB, Ar-Go-Me, S-Ar-Go). Statistical analysis showed that the measurements planned and those obtained after surgery were equivalent in Group D. For Group T, the analysis showed equivalence only for one of the considered measurements (ANB). By comparing the results of the two groups, Group D presented a lower level of error than Group T. Conclusions: Digital surgical planning performed significantly better in terms of accuracy of jaw repositioning than the traditional protocol.

## 1. Introduction

Skeletal class III malocclusions are discrepancies observable in the anteroposterior plane, characterized by a disharmonic relationship between cranial bone structures and the maxillofacial complex, or between the maxillary bones only, resulting in a more mesial position of the mandible compared to the maxilla [1,2,3]. This type of skeletal malocclusion is the result of the deficient development of the maxilla, an excessive development, and/or prognathism of the mandible, or most often by a combination of these skeletal problems [1,3,4,5].

The therapeutic strategy’s definition is related to the patient’s age, severity of the malocclusion, chief complaint, clinical examination, and cephalometric analysis [6]. There are three main treatment options for skeletal class III malocclusion: growth modification (possible only in growing patients), orthodontic camouflage, or combined orthodontic and surgical treatment [5,6]. In non-growing skeletal class III patients that cannot be adequately treated only with an orthodontic therapy, the orthodontic–surgical treatment is the therapeutic approach of choice. Therefore, orthognathic surgery is indicated in the treatment of patients with moderate or severe malocclusions which outweigh the implications for an orthodontic correction only [7,8].

In orthognathic surgery, to obtain a result definable as optimal from an occlusal, functional, and aesthetic point of view, it is essential that the 3D jaw base repositioning occurs in the correct way and the position is defined by the maxillofacial surgeon during the surgical planning. Therefore, the accuracy of the surgical planning method used assumes a fundamental role. This phase has several important functions, such as defining, simulating, and confirming the feasibility of the surgical movements necessary to bring the skeletal bases to the planned position, to obtain the appropriate maxillomandibular and occlusal relationship. Therefore, the surgical planning provides to the dental laboratory the necessary information for the production of the surgical splints, which constitute the intraoperative guide for jaws repositioning into the operating room [9,10,11].

In bimaxillary orthognathic surgery, surgical planning is traditionally performed using plaster models mounted in an articulator, by which the dental technician manually produced the surgical splints [10,12,13]. Technological progress led to the transition to digital methods, based on software that allows the performance of surgical planning on a 3D reconstruction of the patient’s skull, obtained from a 3D radiographic imaging (i.e., cone-beam computed tomography). Furthermore, with CAD/CAM technology, surgical splints can be produced by using rapid prototyping machines, with additive or subtractive techniques [9,11,12].

Recent studies have described the accuracy of digital surgical planning and CAD/CAM technology in the surgical treatment of skeletal malocclusions [14,15,16,17,18]. In 2014, a systematic review of the literature aiming to evaluate the precision of 3D virtual surgical planning in orthognathic surgery was published by Stokbro et al. [11]. The authors analyzed clinical studies on this topic present in the literature. This systematic review asserted that virtual surgical planning appears to be an accurate, reproducible, and reliably transferable to the patient (by surgical splints or intraoperative navigation) method, although at that time there was no scientific basis for affirming that an existing method of virtual planning could guarantee better results than the traditional surgical planning. A subsequent systematic review of the literature, published in 2015 by Haas et al. [19], affirmed that the scientific evidence available in this was of low quality, encouraging the execution of further clinical studies aimed at comparing the computer-assisted surgical planning with the traditional method.

This retrospective observational study aimed to compare the accuracy of jaws repositioning in bimaxillary orthognathic surgery using traditional and digital surgical planning in skeletal class III patients.

## 2. Experimental Section

The study design is retrospective observational on a cohort of 60 skeletal class III adult patients (23 males, 37 females, mean age 22.7 years, standard deviation (SD) 4.8) who underwent an orthodontic–surgical treatment and bimaxillary orthognathic surgery. The patients were treated continuously from 2012 to 2019. All the operations were performed at the Maxillofacial Surgery Department of the San Bortolo Hospital of Vicenza (Vicenza, Italy) by the same maxillofacial surgeon. In 2016 the maxillofacial surgeon started to use the digital procedure.

Patients were selected following predetermined inclusion and exclusion criteria:

- skeletal class III (with ANB ≤ 0 and/or Wits appraisal ≤ −2 mm)
- orthodontic–surgical treatment with bimaxillary orthognathic surgery
- availability of complete treatments records (intraoral and extraoral photographs, dental casts, pre/post-cephalometric analysis, 2D presurgical Visual Treatment Objectives (VTO)) of adequate quality
- negative anamnesis for craniofacial congenital anomalies or syndromes
- negative anamnesis for facial traumas
- no previous orthognathic surgery.

The 60 patients were equally divided into two groups based on the method used for performing the surgical planning (Table 1):

- traditional Group T (11 males and 19 females, mean age 22.8 years, SD 4.9)
- digital Group D (12 males and 18 females, mean age 22.5 years, SD 4.8)

For each patient, a 2D presurgical VTO was prepared by the maxillofacial surgeon for predicting treatment objectives, using the Dolphin Imaging software (Dolphin Imaging & Management Solutions, Patterson Dental Supply Inc).

All the 60 bimaxillary orthognathic surgeries were performed with a maxillary-first procedure.

### 2.1. Traditional Surgical Planning

The traditional procedure consisted of the registration of dental impressions, face-bow transfer, and the mounting of the dental casts onto the articulator. The osteotomies of the two jaws were simulated on the dental casts, based on the objectives defined with the 2D presurgical VTO, and the plaster models reassembled in the articulator with the correct occlusion. To correctly transfer the jaw movements from the 2D VTO to the stone models mounted onto the articulator, dental reference points were used. These were the central incisors and the first molars, both for the maxillary and the mandibular arch. The VTO defined the entity of movements which had to be done in antero–posterior and vertical direction; then movements were transferred on the stone models. All the intermediate and final surgical splints were handmade by the same dental lab.

### 2.2. Digital Surgical Planning

The digital surgical planning was performed using the DDS-Pro software (DDS-Pro version 2.4.0_2020, JST sp, Częstochowa, Poland). The simulation of the two jaw osteotomies and the repositioning of the skeletal bases on the 3D reconstruction of the patient’s skull (obtained by the CBCT performed at the end of the presurgical orthodontic preparation phase) were carried out, as defined with the presurgical 2D VTO. The surgical splints were CAD/CAM: the dental laboratory digitally designed the intermediate and final surgical splints and physically 3D printed with additive technique using a rapid prototyping machine (XFAB 2500PD, DWS Systems, Italy). The material used to produce the splints was DS 3000 resin, created to be used in the oral cavity.

### 2.3. Evaluation of Surgical Accuracy

The correlation between the planned and actual movements measured the accuracy of the two planning methods. The evaluation was performed by comparing determined cephalometric measurements obtained by the 2D presurgical VTO with those obtained in the after surgery’s cephalometry (Roth–Jarabak analysis). All the after surgery’s cephalometry was prepared by the same operator. The cephalometric measurements used for the comparison were: ANB angle, for the evaluation of the skeletal class; SNA and SNB angle, to evaluate the anterior–posterior position respectively of the upper and lower jaw; and Gonial and Articular angles, for the evaluation of verticality.

Data were then collected and organized in a table (Excel, Microsoft Office 365, Microsoft, Redmond, USA).

### 2.4. Statistical Analysis

To verify the hypothesis of equivalence between the planned and obtained post-surgical result, authors set a tolerance margin of 2°. Sample size calculation required 30 cases per group to demonstrate, with a power of 90% and a Type I error of 5%, that the difference between the planned and obtained post-surgical result did not exceed 60% of the SD. The same sample allowed us to demonstrate, with a power of 90% and a Type I error of 5%, a mean difference between traditional and digital surgical planning equal to 85% of the SD. Continuous data were expressed as mean and SD and categorical one as frequency and percentage. The hypothesis of equivalence between the obtained and planned post-surgical results was verified with the TOST test (two one-sided test). The comparison between the traditional and digital methods for surgical planning was performed with the two-tailed Student’s *t*-test. The statistical significance level in all tests was set at a *p*-value ≤ 0.05. The statistical analysis was performed using R 3.5 (R Foundation for Statistical Computing, Vienna, Austria).

## 3. Results

Table 2 shows the initial characteristics of the cases included in the study, the initial ANB angle and Wits appraisal, defined by the pretreatment cephalometric analysis according to Roth–Jarabak. No significant differences emerged between the two groups.

Table 3 shows the difference (in absolute value) between the results obtained using the traditional surgical planning. The statistical analysis suggests an equivalence between the result obtained and planned only for the ANB angle (*p* = 0.003). For the other measures, the existence of equivalence between the obtained and the planned post-surgical result cannot be affirmed. The cephalometric measurement for which the traditional surgical planning showed less equivalence of the result is the Ar-Go-Me angle (*p* = 0.78): an absolute difference higher than 2° of the planned measurement compared to that obtained appeared in 33% of the cases. Both for the values of the SNA angle (*p* = 0.72) and SNB angle (*p* = 0.06), an absolute difference higher than 2° was observed in 23% of cases. All this evidence was considered statistically significant (*p* > 0.05).

Table 4 shows the difference (in absolute value) between the result obtained using digital surgical planning. The statistical analysis suggests equivalence between the obtained and planned post-surgical result for all the measures considered (*p* < 0.05). The cephalometric measurement for which digital surgical planning showed higher equivalence of the result is the S-Ar-Go angle (*p* < 0.0001); in fact, in all of the considered cases, an absolute difference lower than 2° of the planned measurement compared to the obtained with surgery occurred. On the contrary, a lower equivalence of the result was found for the Ar-Go-Me angle, for which the absolute difference between the planned and obtained cephalometric measurement appeared higher than 2° in 10% of the cases. However, this evidence was considered not statistically significant and the statistical analysis showed equivalence of the result for the Ar-Go-Me angle too (*p* < 0.0001).

In general, the difference (in absolute value) between the obtained and planned post-surgical result was more limited with the digital surgical planning compared to the traditional surgical planning. Table 5 shows the comparison between the two surgical planning’s methods.

## 4. Discussion

Traditional surgical planning has been extensively used, studied, and improved over time, and obtained good and reliable results [20,21]. However, model surgery presents some limits and errors that can accumulate during its different phases, both clinical and laboratory, leading to substantial inaccuracies in the final outcome [13,20,21]. Technological progress led to innovations in the treatment and surgical planning phases, allowing progressive movement from traditional methods to more modern and digital techniques. Recent studies described the accuracy of digital surgical planning and CAD/CAM technology in the surgical treatment of skeletal malocclusions [14,15,16,17,18].

The main objective of the present study was to compare the accuracy of jaw repositioning in bimaxillary orthognathic surgery using the traditional and digital methods for surgical planning. In the two methods, the surgical splints were obtained respectively with manual technique and CAD/CAM technology, so the study has indirectly allowed to get some considerations about the accuracy of the two different splints production methods. The choice to focus the analysis on a sample consisting of only skeletal class III patients is motivated by the research of the sample homogeneity. The distribution of the sample based on the mean age at the time of the surgery, the sex and the initial cephalometric measurements (ANB angle and Wits appraisal) was similar in the two groups studied, making the studied sample further homogeneous.

In the present study, only the hard tissues were considered for comparison, as well as only angular cephalometric measurements, as linear measurements were considered more susceptible to errors. The choice to focus the analysis only on hard tissues was justified by the fact that the postoperative soft tissue swelling and their functional adaptation needs about 6–12 months [3,22] from surgery to completely resolve and stabilize. A soft tissue analysis performed on the radiographic documentation of the immediate postoperative period would not have been completely reliable. Therefore, for a precise evaluation of the soft tissues, a radiographic documentation (possibly 3D) of at least 12 months post-surgery would have been necessary, whose execution was not routinely foreseen and, consequently, was not available for all patients. However, taking as the assumption that the position of the soft tissues directly depends on the underlying supportive hard tissues, the extension of the analysis only to the hard tissues was considered sufficient to adequately satisfy the aim of this study.

The first part of the analysis of the obtained data aims to assess the individual accuracy of the two surgical planning methods, comparing them with the post-surgical results. In Group T, the statistical analysis revealed a lack of equivalence between the planned and obtained results for all the cephalometric measurements considered in the comparison, except for the ANB angle (*p* = 0.003). Instead, in Group D, the statistical analysis showed an equivalence all the cephalometric measurements considered for the comparison (*p* < 0.05). The second analysis pursued the main objective of this study, comparing the accuracy of the traditional and digital surgical planning method. The results obtained from the statistical analysis of the data indicated a difference in terms of accuracy between the two surgical planning methods in bimaxillary orthognathic surgery. In fact, for the cephalometric measurements considered for the comparison, the difference in absolute value between the planned and obtained postoperative result was more limited for the digital surgical planning method compared to the traditional. Therefore, we can affirm that digital surgical planning appeared more accurate in programming and predicting the post-surgical result.

The finding of a greater accuracy of digital surgical planning compared to traditional surgical planning has led to two considerations. First, the procedure of surgical preparation with the digital method, based on a dedicated software and virtually performed on the CBCT and virtual models, allows clinicians to define more precisely the exact measurement of the surgical displacements that the maxillofacial surgeon will reproduce in the operating room. Secondly, the CAD/CAM surgical splints are very precise in reproducing the planned skeletal position, consistent with the virtual one, and do not require laboratory procedures.

In the literature, some other studies pursued the same aim [14,20,23]. They demonstrated similar but non-comparable results and conclusions, because they used different operating procedures, software for the preparation and analysis methods. The consideration that can be drawn is that despite the differences previously mentioned, in their respective studies several authors concluded that the digital surgical planning is comparable or more accurate than the traditional method.

A possible limitation of this study was that for the comparison between the planned and obtained post-surgical result, a 2D radiographic documentation was used, instead of a 3D radiographic imaging. The choice was motivated by the fact that postoperative 3D radiographic documentation was not available for all patients, in particular for cases for which surgical planning was performed with the traditional method, that were older and for which the operating procedure did not still provide the collection of 3D radiographic documentation both preoperative and postoperative. For all patients, however, a lateral teleradiograph of the skull was performed to the hospital a few days after surgery.

## 5. Conclusions

This study aimed to compare the accuracy of jaw repositioning in bimaxillary orthognathic surgery using the traditional and digital method for surgical planning in skeletal class III patients. The obtained results showed that the accuracy of jaw repositioning was higher for the group of patients for whom a digital surgical planning was performed. From a clinical point of view, these results appeared useful to demonstrate how the introduction in orthognathic surgery of a digital technology for surgical planning and splints produced with CAD/CAM technology can bring an improvement in the predictability of post-surgical results, compared to traditional surgical planning.

## Figures and Tables

**Table 1:** Distribution of the sample based on the average age at the time of surgery and sex.

	All	Traditional	Digital
Total, *n*	60	30	30
Males, *n*	23	11	12
Females, *n*	37	19	18
Mean age, years (SD)	22.7 (4.8)	22.8 (4.9)	22.5 (4.8)

SD: standard deviation.

**Table 2:** Initial cephalometry.

	All: Average (SD)	Traditional: Average (SD)	Digital: Average (SD)	*p*-Value
*n*	60	30	30	
Wits, mm	−8.9 (4.6)	−9.5 (4.7)	−8.4 (4.5)	0.32
ANB	−2.9 (3.3)	−3.1 (2.8)	−2.6 (3.7)	0.52

ANB: angle between line AN and line BN.

**Table 3:** Traditional surgical planning.

Measure	Difference (in Absolute Value) between the Obtained and Planned Result, Average (SD)	Cases with Absolute Difference > 2°, *n* (%)	Equivalence Test: 90% CI (*p*-Value)
ANB, *°*	1.32 (1.26)	4 (13%)	0.93 to 1.71 **(*p* = 0.003)**
SNA, °	2.30 (2.29)	7 (23%)	1.59 to 3.01 (*p* = 0.72)
SNB, °	2.02 (1.96)	7 (23%)	1.41 to 2.63 (*p* = 0.06)
Ar-Go-Me, °	2.27 (1.87)	10 (33%)	1.69 to 2.85 (*p* = 0.78)
S-Ar-Go, °	1.62 (1.65)	4 (13%)	1.11 to 2.24 (*p* = 0.11)

SNA: angle between line SN and line AN; SNB: angle between line SN and line BN; Ar-Go-Me: Gonial angle (angle between line Ar-Go and line Go-Me); S-Ar-Go: Articular angle (angle between line S-Ar and line Ar-Go).

**Table 4:** Digital surgical planning.

Measure	Difference (in Absolute Value) between the Obtained and Planned Result, Average (SD)	Cases with Absolute Difference > 2°, *n* (%)	Equivalence Test: 90% CI (*p*-Value)
ANB, °	0.77 (0.62)	1 (3%)	0.58 to 0.96 **(*p* < 0.0001)**
SNA, °	0.96 (0.77)	1 (3%)	0.72 to 1.20 **(*p* < 0.0001)**
SNB, °	0.77 (0.71)	1 (3%)	0.55 to 0.99 **(*p* < 0.0001)**
Ar-Go-Me, °	0.96 (0.91)	3 (10%)	0.68 to 1.24 **(*p* < 0.0001)**
S-Ar-Go, °	0.54 (0.50)	0 (0%)	0.39 to 0.70 **(*p* < 0.0001)**

**Table 5:** Comparison between traditional and digital surgical planning.

Measure	Difference (in Absolute Value) between the Obtained and Planned Result, Average (SD)			
	Digital Surgical Planning	Traditional Surgical Planning	Mean Difference (95% CI)	*p*-Value
ANB, °	0.77 (0.62)	1.32 (1.26)	−0.55 (−1.07 to −0.03)	**0.04**
SNA, °	0.96 (0.77)	2.30 (2.29)	−1.34 (−2.24 to −0.45)	**0.004**
SNB, °	0.77 (0.71)	2.02 (1.96)	−1.25 (−2.02 to −0.48)	**0.002**
Ar-Go-Me, °	0.96 (0.91)	2.27 (1.87)	−1.31 (−2.07 to −0.55)	**0.001**
S-Ar-Go, °	0.54 (0.50)	1.62 (1.65)	−1.08 (−1.72 to −0.44)	**0.002**
